# Dysphagia, an uncommon initial presentation of sarcoidosis

**DOI:** 10.1016/j.rmcr.2022.101647

**Published:** 2022-04-04

**Authors:** Navjot Somal, Ravi Karan, Aarti Maharaj, Jeff Halperin, Brent Boodhai, Jeffrey Lipton, Daniel J. Masri, Kamlesh Kumar

**Affiliations:** Maimonides Medical Center, USA

## Abstract

Sarcoidosis is a systemic inflammatory disease of unknown etiology with a myriad of clinical presentations depending on the organ systems involved. Neurosarcoidosis is an uncommon entity which is characterized by non-caseating granuloma infiltration of the central nervous system. Dysphagia in sarcoidosis is even more uncommon, and can involve one or more pathophysiological mechanisms: central nervous system involvement (cranial nerves associated with swallowing), lower motor neuron involvement (invasion of the enteric nervous plexus), direct muscle infiltration (invasion of the skeletal muscle portion of the esophagus and posterior pharynx), or mechanical obstruction (extrinsic compression by mediastinal lymph nodes). We report a case of a middle-aged woman presenting with severe dysphagia due to neurosarcoidosis which markedly improved after starting corticosteroids. The purpose of this case report is to highlight an atypical presentation of this disease.

## 1 Case presentation

A 49-year-old woman from Bangladesh presented with a three-month history of progressive dysphagia to solids and liquids, dry cough, malaise, and associated 30-pound unintentional weight loss. She was admitted to multiple hospitals due to similar complaints of dysphagia and progressively worsening symptoms, but no formal diagnosis was made. She denied any fevers or hemoptysis. Endoscopic gastroduodenoscopy (EGD) performed in the outpatient setting two weeks prior to admission demonstrated a peptic ulcer but was otherwise unremarkable. She was also diagnosed with right-sided facial nerve palsy a month prior to her admission and was treated with a two-week course of corticosteroids. Her symptoms relapsed after discontinuation of steroid therapy.

The patient's vital signs were stable throughout her admission. On examination, there was significant cachexia and new left facial weakness in a lower motor neuron pattern consistent with facial nerve palsy. She was alert and displayed no signs of cognitive impairment. She was unable to completely close the right eye. There was absence of conjunctival chemosis, proptosis, or periorbital edema. Uveitis was ruled out by an ophthalmologic exam. There was decreased sensation in right V1–V3 distribution, hearing was reduced on right compared to left to finger rub, decreased sensation in the right oropharynx, symmetric rise of the palate, and her tongue appeared midline on protrusion. Cranial nerves were otherwise grossly intact. She had decreased muscle bulk throughout and 5/5 strength in the arms. In the legs, she had bilateral 4/5 proximal strength and 5/5 distal strength. She had normal reflexes in both upper and lower extremities.

**Fig. 1:**
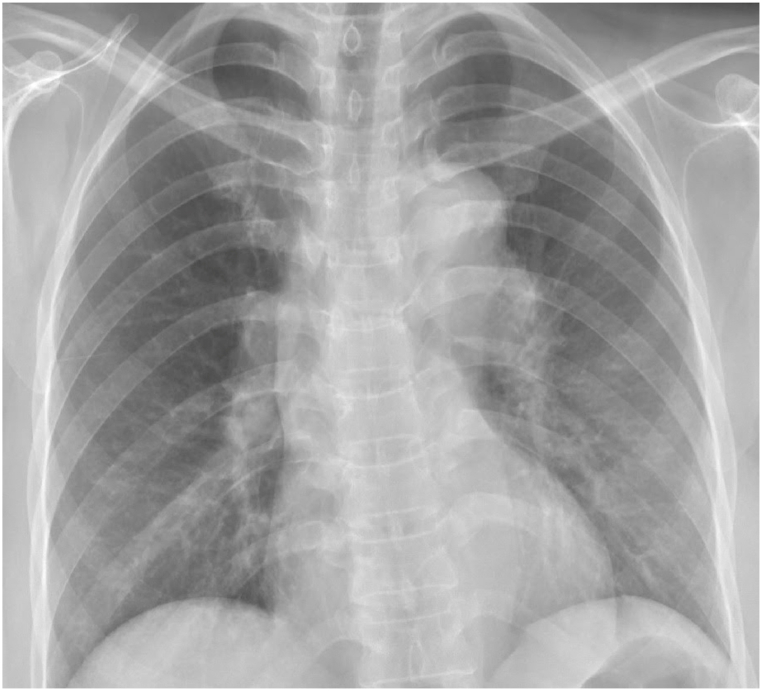
Chest radiograph demonstrating bilateral hilar adenopathy.

**Fig. 2:**
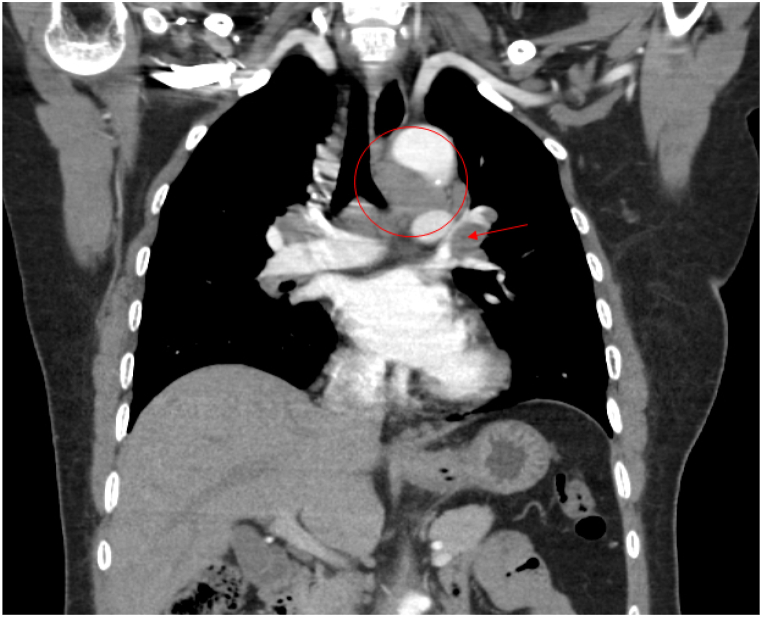
Longitudinal section of computed tomography scan of the chest showing mediastinal lymphadenopathy abutting the esophagus.

The patient's laboratory investigations revealed BUN 3.97 mg/dl and Na 132 mmol/L. She was initially placed on airborne precautions, given constitutional symptoms and positive Quantiferon tuberculin testing, which were subsequently discontinued after obtaining sputum samples that were negative for acid fast bacilli. Chest radiograph demonstrated hilar prominence but no focal airspace opacities (Fig. 1). Computed tomography (CT) of the chest was significant for interval development of extensive bilateral paratracheal, subcarinal, and bilateral hilar lymphadenopathy (Fig. 2) consistent with Garland's triad.

A fiber-optic endoscopy was performed to evaluate dysphagia which revealed immobility of the right vocal fold, significant pooling of secretions, hooding of arytenoids with erythema and edema, and oropharyngeal dysphagia. Initial videofluoroscopic study revealed indentation of the hypopharynx in the region of the cricopharyngeal muscle. The study was aborted given inability to adequately clear moderate puree residue in the pyriform sinuses and proximal esophagus, as well as significant coughing, gagging, and hocking in the absence of aspiration. She was placed on aspiration precautions and enteral feeding was started via nasogastric tube.

Due to bilateral facial nerve palsy, a lumbar puncture was performed which showed an opening pressure 15 mmHg, white cell count of 18 [0–10/UL] with predominant lymphocytes 93% [40–80%], red blood cell of 3, protein 66 [20–100 mg/dl], Glucose 62 [40–170 mg/dl]. Oligoclonal Banding was absent. Various serum and cerebrospinal fluid (CSF) studies were done to evaluate neoplastic, paraneoplastic, infectious, and inflammatory etiologies but were all unremarkable. Serum HIV 1/2 EIA, serum and CSF VDRL, serum HSV 1/2 PCR, serum EBV panel, serum and CSF Lyme disease total Antibody IgG/IgM were negative. Angiotensin Converting Enzyme in serum and CSF were 65 [14–82 U/L] and 2.1 [0.0–2.5 U/L], respectively. Although serum ACE has historically been the most widely known serum biomarker in sarcoidosis, its sensitivity ranges 22–86% and specify 54–95% making their clinical utility a matter of debate [1].

**Fig. 3:**
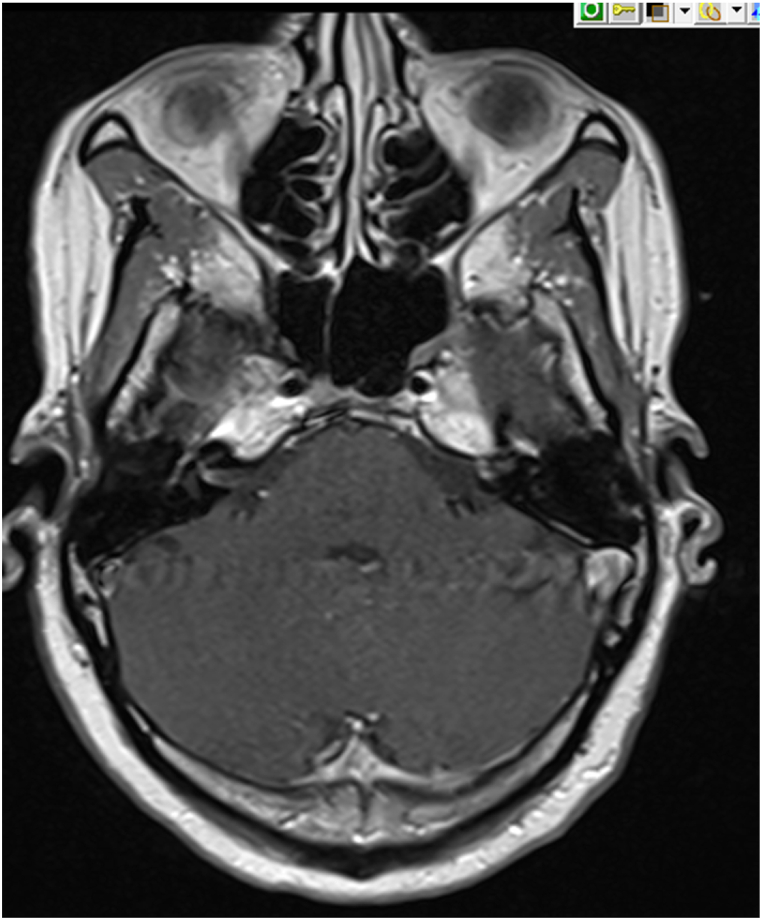
Thin cut Axial T1 postcontrast imaging through the internal auditory cancals demonstrates enhancement of the bilateral 7th/8th cranial nerve complexes.

**Figs. 4:**
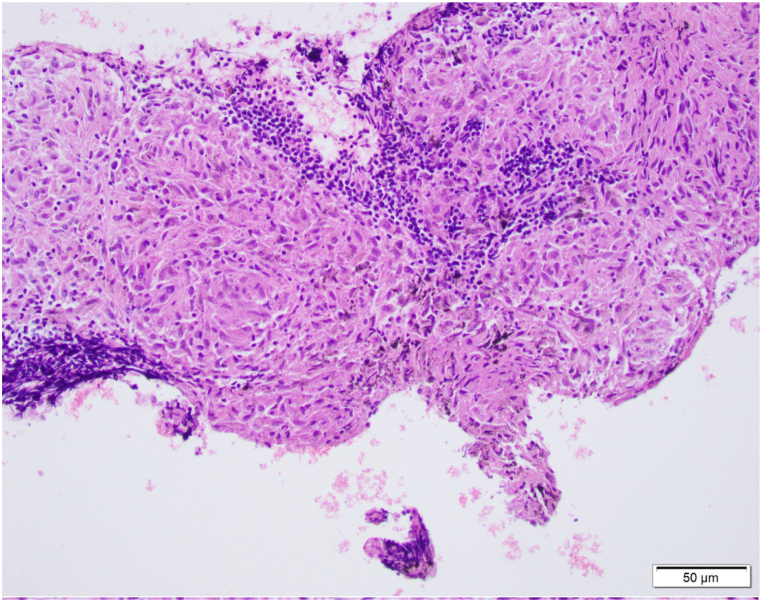
EBUS-TBNA of a lymph node (L4: left lower paratracheal lymph node) revealed noncaseating granulomas composed of epithelioid histiocytes. The results of tissue smear, culture, for acid fast bacilli, fungi, and flow cytometry for lymphoproliferative disorder were negative. EBUS-TBNA: endobronchial ultrasound-guided transbronchial needle aspiration (Hematoxylin and Eosin staining, × 200 magnification).

**Fig. 5:**
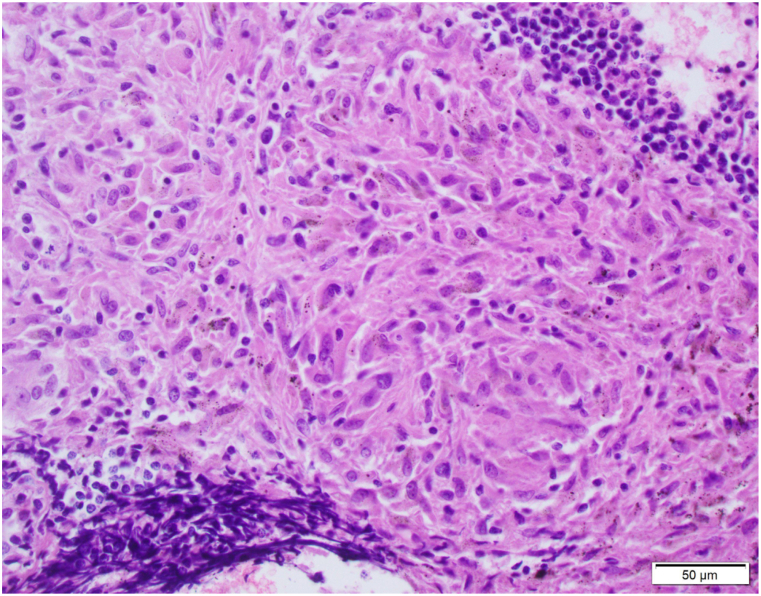
EBUS-TBNA of a lymph node (L4: left lower paratracheal lymph node) revealed noncaseating granulomas composed of epithelioid histiocytes. The results of tissue smear, culture, for acid fast bacilli, fungi, and flow cytometry for lymphoproliferative disorder were negative. EBUS-TBNA: endobronchial ultrasound-guided transbronchial needle aspiration (Hematoxylin and Eosin staining, ×400 magnification).

Magnetic resonance imaging of the brain with and without contrast showed enhancement of the seventh and eight cranial nerves concerning for granulomatous disease or lymphoma (Fig. 3). MRI of the l-spine showed no acute fractures, no compression of the conus or causa equine and no high grade central canal or neural forminal stenosis. Since the CT scan of the chest showed significant lymphadenopathy, she underwent endobronchial ultrasound (EBUS) guided fine needle aspiration (FNA) with multiple lymph node biopsies, which revealed non-caseating granulomas without malignant cells (Figs. 4, Fig. 5). Further testing and interpretation by the was significant for the absence of acid-fast bacilli by AFB stain, absence of fungi by GMS stain, and absence of immunophenotypic evidence of a lymphoproliferative disorder by flow cytometry.

The patient was diagnosed with probable neurosarcoidosis given the clinical presentation, laboratory evidence of CNS inflammation, MRI evidence of granulomatous involvement of cranial nerves VII and VIII, and extra-neural lymph node biopsy suggesting of sarcoidosis with positive histology [Fig. 1] as per the Neurosarcoidosis Consortium Consensus Group [2]. Neural tissue biopsy was not done to confirm the diagnosis in this patient since it is considered an invasive procedure and enough evidence was obtained to initiate treatment without further delay. The patient was immediately started on intravenous methylprednisolone 40mg every 12 h. In light of the need for a long-term course of high dose corticosteroids, treatment for latent tuberculosis and pneumocystis carinii pneumonia prophylaxis were also commenced. She had significant subjective improvement in dysphagia and musculoskeletal symptoms within the first week of treatment. There was modest improvement in the left facial nerve palsy and resolution of lower extremity weakness. Repeat videofluoroscopic study performed one week after steroid therapy showed improved pharyngeal motility. Diet was slowly advanced and intravenous corticosteroid was transitioned to oral glucocorticoid. Follow-ups with rheumatology, infectious disease, neurology, and pulmonology were provided.

## 2 Discussion

Sarcoidosis is a systemic inflammatory disease characterized by the presence of non-caseating granuloma in the affected organ. While there have been substantial advances in our understanding of the pathogenesis, the etiology of sarcoidosis still remains obscure, likely due to the heterogeneity of the disease and its unpredictable clinical course. Intra-thoracic involvement is the hallmark of sarcoidosis and is present in over 90% of patients [3]. The skin, eye, liver, and peripheral lymph nodes are the next most commonly clinically involved organs in most series, with the frequency of involvement ranging from 10 to 30% [4]. However, gastrointestinal involvement is a rare manifestation with the prevalence of clinically relevant disease present in 0.1–0.9% of patients [5,6]. Furthermore, esophageal involvement is an extremely uncommon presentation with only a few cases reported. Esophageal involvement predominantly presents with dysphagia (91.3%) with the lower esophagus being most susceptible. It can also manifest as weight loss, as demonstrated in this case, as well as abdominal pain, odynophagia, dysphonia, or anemia [7].

Esophageal compromise occurs through direct and indirect mechanisms. It can also be categorized according to the layer of esophageal involvement (superficial mucosal, muscular layer, neuronal involvement, extrinsic compression). The superficial mucosal involvement may present as several discrete gray plaque-like lesions or mucosal hyperemia and nodularity [8]. Our patient had a recent outpatient endoscopy without evidence of the aforementioned, essentially ruling out superficial mucosal involvement as the pathological mechanism behind her dysphagia.

There have also been reports of myopathic involvement of the skeletal muscle portion of the esophagus and posterior pharynx. Nishikubo et al. reported a case of a 70-year-old woman presenting with a chronic dry cough. Videofluorography showed marked stenosis posteriorly at the level of pharyngoesophageal junction. She subsequently underwent cricopharyngeal myotomy with histology of the tissue revealing non-caseating granulomas [9]. Initial videofluoroscopic study of our patient revealed indentation of the hypopharynx in the region of the cricopharyngeal muscle indicating potential myopathic granulomatous infiltration. However, her symptoms showed marked improved with steroid therapy and did not require any surgical intervention.

Direct involvement of the enteric nervous plexus can cause dysphagia, and the clinical picture can mimic that of achalasia. Previous studies have reported that the nervous system is involved in approximately 5% of cases, and in postmortem examination, the rates are as high as 15–27% [10]. Histopathological studies often show diffuse inflammatory infiltration of the myenteric plexus with complete demyelination, loss of axons, and active degeneration [11].

Sarcoidosis is well-known to affect the cranial nerves most commonly the facial and optic nerve, followed by collective involvement of cranial nerves IX, X, XI [12]. Our patient presented with bilateral facial nerve palsy which prompted high clinical suspicion for additional cranial nerve involvement that could be contributing to her dysphagia. The complex mechanism of swallowing involves cranial nerves V, VII, IX, X, XII, however, to what extent any or all of these nerves and associated muscles are affected could not be quantified aside from physical exam and imaging. Decreased sensation of right V1–V3 distribution, facial nerve palsies, and decreased sensation of right oropharynx suggested CN V, VII, IX involvement, respectively. MRI brain showed enhancement of only the seventh and eighth cranial nerves with normal appearances of cranial nerves IX, X and XII making dysphagia secondary to cranial nerve involvement a possibility in this case.

Dysphagia can also be explained as a consequence of direct extrinsic compression from enlarged mediastinal lymph nodes. In a review of literature published until September 2016, four cases of dysphagia secondary to extrinsic node compression of the esophagus were identified. Such cases showed resolution of dysphagia with oral corticosteroids [13]. Although extremely rare, a case of esophageal perforation was reported as a consequence of mediastinal lymph node hypertrophy causing esophageal erosion, necrosis and ultimately perforation [14]. In this case report, extensive paratracheal, subcarinal and bilateral hilar adenopathy were noted on imaging and histology of the lymph nodes revealed non-caseating granulomas (Figs. 4, Fig. 5). Her symptoms improved significantly with intravenous administration of corticosteroids and a follow-up chest radiograph after steroid therapy depicted improvement of hilar lymphadenopathy. Although not definitive, it does suggest that direct extrinsic compression may have contributed to her symptoms.

Due to its rarity, there have been no formal clinical trials comparing the efficacy of the different treatment modalities for the management of esophageal sarcoidosis. In a review of 43 cases, Brito et al. reported corticosteroid therapy to be the first-line treatment in over 40% of cases of sarcoidosis with esophageal involvement with excellent clinical response. Patients with sarcoidosis often require prolonged therapy, especially with severe disease and often require steroid-sparing medication to avoid relapse. The most frequently recommended second line therapy used is methotrexate based on its well-established side effect profile and efficacy in autoimmune disease. It has been shown to be an effective steroid-sparing agent in two clinical intervention studies (one randomized and one non-randomized), where patients showed improvement in vital capacity or symptomatic organ dysfunction while concurrently tapering down on steroids [15].

One of the most recent innovations in the management of sarcoidosis is the use of monoclonal antibodies against TNF-alpha such as infliximab and adalimumab. In one double-blind, placebo-controlled Phase II trial of 138 patients with chronic pulmonary sarcoidosis, infliximab significantly increased the percent of predicted FVC compared to baseline by 2.5%, whereas placebo did not [15].Surgical intervention may be required in patients with esophageal wall muscular involvement, suspected achalasia, esophageal strictures or rare life-threatening complications such as perforation [16]. Botulinum toxin infiltration has also been documented as a temporary measure in patients with achalasia prior to administration of definite therapy with corticosteroids or surgery [17].

## 3 Conclusions

The diagnosis of sarcoidosis with esophageal involvement poses a diagnostic challenge due to its rarity and variable presentation. Despite its infrequency, the diagnosis is clinically relevant since progression can lead to malnutrition and debilitating disease or rarely become life-threatening in the form of esophageal perforation. Therefore, as clinicians managing this complex disease, it is imperative to maintain a high index of suspicion for sarcoidosis-induced dysphagia in the appropriate setting.

## Declaration of competing interest

There are no conflicts of interest that need declaration for this case report.
